# Testing for top‐down cascading effects in a biomass‐driven ecological network of soil invertebrates

**DOI:** 10.1002/ece3.6408

**Published:** 2020-06-18

**Authors:** Erminia Conti, Letizia Stella Di Mauro, Alessandro Pluchino, Christian Mulder

**Affiliations:** ^1^ Department of Biological, Geological and Environmental Sciences University of Catania Catania Italy; ^2^ Department of Physics and Astronomy "Ettore Majorana" University of Catania Catania Italy; ^3^ INFN Unit of Catania Catania Italy

**Keywords:** ecological network, functional biodiversity, lotka–volterra, multiple prey–predator model, soil food webs, temporal simulation

## Abstract

1. To investigate the structural changes of a food‐web architecture, we considered real data coming from a soil food web in one abandoned pasture with former low‐pressure agriculture management and we reproduced the corresponding ecological network within a multi‐agent fully programmable modeling environment in order to simulate dynamically the cascading effects due to the removal of entire functional guilds.
2. We performed several simulations differing from each other for the functional implications. At the first trophic level, we simulated a removal of the prey, that is, herbivores and microbivores, while at the second trophic level, we simulated a removal of the predators, that is, omnivores and carnivores. The five main guilds were removed either separately or in combination.
3. The alteration in the food‐web architecture induced by the removal of entire functional guilds was the highest when the entire second trophic level was removed, while the removal of all microbivores caused an alteration in the food‐web structure of less than 5% of the total changes due to the removal of opportunistic and predatory species.
4. Omnivores alone account for the highest shifts in time of the numerical abundances of the remaining species, providing computational evidence of the importance of the degree of omnivory in the stabilization of soil biota.

## Graphical abstract

Sketch of the empirical soil network used to simulate the cascading effects of guild removals in our prey–predator relationships.

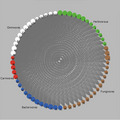

## 1 INTRODUCTION

Interacting species are embedded within complex systems, and according to their traits, the species determine the architecture of the entire food web (Brose et al., 2019; Potapov, Brose, Scheu, & Tiunov, 2019). Despite the huge amount of literature on a catastrophic species decline (IPBES, 2019), experimental evidence on weakened functional groups (less individuals, less species, and less guilds) is lacking from the soil biota. That is surprising, as the functional loss due to human impact would strongly erode the health of entire agroecosystems and deserves more attention. For instance, an inverse correlation between aboveground farming intensity and the belowground functional diversity is known (Mulder, De Zwart, Van Wijnen, Schouten, & Breure, 2003; Mulder, Cohen, Setälä, Bloem & Breure, 2005) and makes some assumptions on any constant biomass distribution restrictive. Hunt et al. (1987) assumed that biomass inputs exactly balance biomass outputs at all times in each soil compartment of the detrital food web. But this steady‐state assumption not always holds, as the biomass decreases of microorganisms, microfauna and mesofauna indicate negative effect on the soil buffer capacity (Hunt & Wall, 2002; Wall, Nielsen & Six, 2015) due to an increased pressure, or even soil exploitation, caused by intensive management practice (Mulder et al., 2011; Mulder, Den Hollander, & Hendriks, 2008).

There is a consensus regarding the evidence that any loss of keystone species severely disrupts ecosystem functioning (IPBES, 2019), but the functional impacts on the food‐web architecture of such decimations are almost unknown. Functional guilds are keystone units and can be defined as trait‐driven groups of species with key roles in community architecture and therefore ecosystem functioning (Mouquet, Gravel, Massol, & Calcagno, 2013; Power et al., 1996). Recently, Brose et al. (2019) pointed out the importance of prey–predator relationships, demonstrating that metabolic rate and functional traits of predatory species are more important in determining the interactions between the weight of the prey and the weight of the predator. Despite this recent meta‐analysis, it seems often difficult to extrapolate such outcomes to a much wider context, like behavioral ecology, ecosystem services, or ecosystem functioning (QUINTESSENCE, 2016). Hence, such an extrapolation has to rely upon undiluted mathematical evidences (Bourne, Brenner, & Eisen, 2005; Cohen, 2004).

Mathematically, food webs can be seen as complex ecological networks (Pascual & Dunne, 2005), that is, as graphs whose nodes represent the species present in the ecosystem and whose edges, or links, symbolize the prey–predator relationships between the various species. In particular, a food web is represented by a directed graph, because the connections are expressed by oriented links, which connect the prey/resource to the predator/consumer. In other words, the direction of a given link follows the flow of energy that, through predation, passes from the prey to its predator. Each node/species will be therefore characterized by a degree that defines the total number of (ingoing and outgoing) links connecting it to the other nodes. These links reflect the architecture of any food web and characterize the energy flux across trophic levels and therefore ecosystem functioning as a whole.

Big data at all biological scales became a central feature of research and discovery in the life sciences (Bourne et al., 2005; QUINTESSENCE, 2016). Our aim is to propose here a simple method to quantify how disproportionate the impact of less functional diversity can be and to illustrate its application with one real soil food web. In particular, we rebuilt the considered ecological network (Mulder & Elser, 2009) within a fully programmable multi‐agent environment (Wilensky, 1999) in an attempt to figure out the cascading effects by weakening the functional diversity according to the of soil invertebrates through a simulated removal of entire functional guilds.

## 2 MATERIALS AND METHODS

### 2.1 Data sampling

We used a reference data set of empirically observed soil invertebrates in the sandy soils of one Dutch pasture with former low‐pressure management (Mulder & Elser, 2009). Three replicate samples of about 5 m^2^ from the upper 10 cm of soil for the fauna were taken. Bulk samples of 50 soil cores (diameter 2.3 cm) were used to extract the microfauna, and two soil cores (diameter 5.8 cm) were used to extract the mesofauna.

Extraction of free‐living nematodes was performed within one week of core sampling using Oostenbrink funnels, and all the elutriated nematodes were collected; ecto‐ and endoparasitic nematodes were recovered with centrifugal flotation. All nematode individuals were counted, and ~150 randomly chosen specimens were identified and measured under a light microscope (Mulder & Vonk, 2011). Enchytraeid worms (Oligochaeta: Enchytraeidae) were sampled by wet extraction and microarthropods (Acarina and Collembola) by dry extraction (Cohen & Mulder, 2014). In both sampling protocols, the heat was increased gradually with incandescent bulbs, and the invertebrates escaped by moving downward. For enchytraeids and microarthropods, the abundances for 1 m^2^ × 10 cm depth were derived from the surface and the bulk density of the soil samples (Mulder et al., 2011).

All these organisms live in a dark and intricate world, interacting in a detrital food web and in close contact with the soil. Each organism has its own trait‐driven function in soil biota, giving the soil its exclusive properties, and an interaction matrix was created based on an inventory of multitrophic interactions of soil food webs that provide all links consistent with literature‐derived guilds (Mulder & Elser, 2009: their table S2). Despite the observation that energetic equivalence rule is rarely supported within local communities (Morlon et al., 2009), our reference soil food web was particularly stable according to the Eltonian rule (Elton, 1927). The lumped dry weight of all the sampled invertebrates of the first trophic level was exactly 10.28 times the lumped dry weight of all the sampled invertebrates of the second trophic level, as expected according to the energetic equivalence rule (Mulder & Elser, 2009).

### 2.2 Analysis of the food web: a modified Lotka–Volterra model

In contrast to previous studies on simulated *species* extinction (Ives & Cardinale, 2004), we want to disentangle the cascading effects of the removal of selected functional *groups* (here, entire guilds of soil invertebrates identified at genus or family). Using the data discussed in the previous paragraph, a real food web was built (Table S1) within NetLogo (Wilensky, 1999). For these 62 taxa (hereafter, just *species*), we know the abundance *X_i_*, the body mass *M_i_*, the biomass *B_i_* (given by *B_i_ = X_i_ M_i_*), and the value of the growth rate *r_i_* in condition of interaction, with *i = 1, 2, ..., n*. All identified soil invertebrate “species” fell into five main guilds, and the independent trophic links among guilds (from any possible prey to its consumer) were inferred from published literature. The complete inventory of multitrophic interactions consistent with literature‐derived guilds is fully downloadable from Mulder and Elser (2009). In Figure S1, we show this ecological network, with the numbered nodes/species placed in a circular layout, where each group of species is distinguished by a different color—as explained in detail in Table S1. The size of each node in online Figure S1 is proportional to the base‐10 logarithm of the abundance of the corresponding species (Log *X_i_*).

As multitrophic interactions between basal consumers and allochthonous resources are donor‐controlled, that is, according to Polis, Anderson, and Holt (1997) “consumers benefit from but do not affect resource renewal rate,” we postulated constant allochthonous resources. Our lemma is therefore that the faunal populations have an unlimited resource supply of bacteria, fungi, and roots. This is because we are focusing only on the prey–predator interactions among invertebrates and not on all the resource–consumer interactions occurring in soil biota. Hence, we keep in our simulations microbial and plant biomasses constantly available for grazing by basal (specialized) species and nonbasal (omnivore) species (cf. Hunt et al., 1987; Polis et al., 1997). Then, our assumptions will be:

- In the absence of predators, the population of the prey would grow proportionally to its size.
- In the absence of prey, the population of the predator would decline proportionally to its size, meaning extinction of that population.
- When both predator and prey are present, the interspecific effect of the predation has to be represented as a decrease in the population of the prey and an increase in the population of the predator (Supporting information).

When in an ecosystem there are more than one prey and one predator species, assuming a fixed amount of foraging effort, the Lotka–Volterra equations can be extended as in Hodzic, Selman, and Hadzikadic (2016):

(1) $$\frac{dX_{i}}{\mathrm{dt}}=X_{i}\left[A_{i}+\sum_{j}A_{\mathrm{ij}}X_{j}\right]$$

where *i*, *j = 1, 2, ..., n*, being *n* the total number of species in the ecosystem. Coefficient *A_i_* is the intrinsic growth rate of the *i*‐th species, while coefficients *A_ij_* in the summation take into account the effect of the predation. We would like that the term within parenthesis can be interpreted as a sort of generalized growth rate for species *X_i_,* which considers the interaction of *X_i_* with the other species. We can therefore build a community matrix **A**, relative to the ecosystem, whose elements *A_ij_* weigh the effect of predation between pairs of species:

$$A=\left[\begin{array}{cccccc} A_{11} & A_{12} & \ldots & A_{1j} & \ldots & A_{1n}\\ A_{21} & A_{22} & \ldots & A_{2j} & \ldots & A_{2n}\\ \ldots & \ldots & \ldots & \ldots & \ldots & \ldots \\ A_{i1} & A_{i2} & \ldots & A_{\mathrm{ij}} & \ldots & A_{\mathrm{in}}\\ \ldots & \ldots & \ldots & \ldots & \ldots & \ldots \\ A_{n1} & A_{n2} & \ldots & A_{\mathrm{nj}} & \ldots & A_{\mathrm{nm}} \end{array}\right].$$

Hence, *A_ij_ ≠ 0* when species *i* and *j* are linked by predation phenomena (in particular, *A_ij_* > 0 when species *i* preys species *j,* and *A_ij_* < 0 when species *j* preys species *i*), while *A_ij_ = 0* when species *i* and *j* do not have any predation connection. Nonzero diagonal elements *A_ii_* represent the phenomenon of cannibalism when an individual of species *i* preys on another individual of the same species.

Our starting point is the logistic equation (Supporting information), which takes into account the intraspecific regulation due to the presence of limited resources, applied here to the abundance *X_i_* of each of the 62 species as in Kondoh (2005):

(2) $$\frac{dX_{i}}{\mathrm{dt}}=r_{i}X_{i}\left(1-\frac{X_{i}}{K_{i}}\right)\;\left(i\;=\;1,\;2,\;...,\;n\right)$$

where *r_i_* and *K_i_* are, respectively, the growth rate and the carrying capacity of the *i*‐th species. In order to adapt these equations to real data, we considered that, in addition to the intraspecific interactions, also interspecific interactions occur among species. For this reason, the growth rate *r_i_* must also consider the effect due to predation. According to the prescription of Equation (1), we assume that, in the presence of interaction, *r*_i_ is expressed as follows:

(3) $$r_{i}=r_{i0}+\sum_{j}A_{\mathrm{ij}}X_{j}\;\;\;\;\;\left(i\;=\;1,\;2,\;...,\;n\right)$$

where *A_ij_* are the elements of the community matrix **A,** and $r_{i0}$ is the growth rate of the *i*‐th species in the absence of interaction. By combining the Equations (2) and (3), one finally gets:

(4) $$\frac{dX_{i}}{\mathrm{dt}}=\left[r_{i0}+\sum_{j}A_{\mathrm{ij}}X_{j}\right]X_{i}\left(1-\frac{X_{i}}{K_{i}}\right)\;\;\;\;\;\left(i\;=\;1,\;2,\;...,\;n\right)$$

As we have already seen, in general the elements *A_ij_* of the community matrix weigh the food interaction among pairs of species (*A_ij_ = 0* when species *i* and *j* do not have any predation connection). In this study, we decided to link these weights to the biomasses of the prey species. Such a biomass‐driven perspective, in fact, focuses on groups of species and on the lumped biomass values of the populations that constitute that functional group (Moore & De Ruiter, 2012).

Let us consider, for example, a generic node/species *i* with degree 2 which is, simultaneously, a predator for a given node/species *m* (therefore, an ingoing link will exist from node *m* toward node *i*) and a prey for another node/species *l* (in this case, a directed outgoing link will exist from node *i* toward node *l*). In this case, we will define the coefficient *A_im_* as the following ratio:

(5) $$A_{\mathrm{im}}=\frac{B_{m}}{\sum_{k}B_{k}}$$

where at the numerator there is the biomass *B_m_* of the prey species *m* and at the denominator the summation of the biomasses of all the prey species of node *i* (included *B_m_*): The coefficient is positive because the flux of energy goes from *m* to *i*; therefore, after an encounter, species *i* will have an increase in abundance. Similarly, we defined the coefficient *A_il_* as follows:

(6) $$A_{\mathrm{il}}=-\frac{B_{i}}{\sum_{h}B_{h}}$$

where at the numerator there is the biomass *B_i_* of the prey species *i* and at the denominator the summation of the biomasses of all the prey species of node *l* (included *B_i_*). In this case, the coefficient is negative because the flux of energy goes from *i* to *l*; consequently, after an encounter, species *i* will have a decrease in abundance. The rationale behind these definitions—which, of course, can be applied to node/species with any degree—is that, when a predator has a diet based on a few prey species, he will consume a greater quantity of each of them depending on their single biomass in relation to the total biomass of its prey species. If, on the contrary, the same predator preys many species, it will consume a smaller quantity of each of them in relation to their biomass compared to the total.

In order to proceed with the calculation of the abundances through Equation (4), we should know the term *r_i0_* which can be inferred from real data by making the following assumption. At the time of sampling, the system was—according to Eltonian rules—in a state of stability in which the abundance of each species, in the presence of interactions with all the other ones, had reached its carrying capacity; thus, *X_i_ = K_i_*. Therefore, according to the Equation (3), it is possible to obtain the value of the net growth rate without interaction, *r_i0_*, starting from that one measured when there is interaction, *r_i_*, considering all the species in their stationary state *K_i_*:

(7) $$r_{i0}=r_{i}-\alpha \sum_{j}A_{\mathrm{ij}}K_{j}\;\;\;\;\left(i\;=\;1,\;2,\;...,\;n\right)$$

The parameter *α* is a coupling coefficient that can be considered as a measure of the interaction strength of a given species within the rest of the food web, and it is chosen so that the carnivores and part of the omnivores (species 57, 58, 59, 60 and 61 of the Table S1) have a negative *r_i0_*. In fact, in the absence of interaction between species (thus without possibility of predation), carnivores must have a negative growth rate. The same happens for omnivores whose diet is composed of animals rather than plants.

Considering our lemma (we kept bacteria, fungi, and roots constantly available to soil invertebrates) and under the further plausible assumption that, in the absence of interaction among species, the carrying capacity of each species, say *K_i_*_0_, would be greater than the same one in the presence of interaction, that is, *K_i_*, we postulate that:

(8) $$K_{i0}=K_{i}+\frac{K_{i}}{2}$$

Notice that, despite this prescription, in the absence of interaction, species with *r_i0_* < 0 will tend to extinction.

Summarizing, we can effectively rewrite our dynamical equations as follows:

(9) $$\frac{dX_{i}}{\mathrm{dt}}=\left[r_{i0}+\beta \alpha \sum_{j}A_{\mathrm{ij}}X_{j}\right]X_{i}\left(1-\frac{X_{i}}{K_{i}+\left(1-\beta \right)\frac{K_{i}}{2}}\right)\;\;\;\;\;\;\;\;\left(i\;=\;1,\;2,\;...,\;n\right)$$

with *β = 0* in the case in which there is no interaction and *β = 1* in the case in which there is interaction, of strength *α*, between the species.

Equation (9), applied to each node of our ecological network, allows us to simulate the dynamical evolution of the system in several representative scenarios, where different kinds of perturbations will be realized in order to study the reaction of the species. Notice that, despite Equation (8), in the absence of interaction(s), species with *r*_i0_ < 0 will tend to extinction as expected. All the simulations were done by choosing the initial abundance of the species in the interval:

(10) $$X_{i}\left(0\right)\in \left[K_{i}-\frac{K_{i}}{2};K_{i}\right]$$

so that they cannot exceed their carrying capacity. For each scenario, starting from the initial conditions (10), at each time step the populations *X_i_*(*t*) of all species are updated by numerically integrating equation (9) until the system has reached a condition of stability. Notice that a variation of *X_i_*(*t*) for a given species implies a variation of its biomass *B_i_*(*t*) = *M_i_ X_i_*(*t*). Then, depending on the chosen scenario, we forced the removal of a certain number of species in the following way. After 100 time steps, enough for reaching a stationary state, that is, for the populations of all the species to have reached their initial carrying capacity *K_i_*, the abundances of the species we have chosen to remove are decreased exponentially over time.

In turn, this can induce cascading effects on some of the other species, according to the following rules: When the abundances of carnivores or omnivores start to decrease in time, it is reasonable to assume that a corresponding increase in the carrying capacity *K_i_* of their prey will occur. The latter can be obtained starting from the potential variation of the biomass of the *i*‐th prey at time *t,* that is calculated as the product of its own current biomass *B_i_*(*t*) times the ratio between the total biomass of dead predators and the total biomass of all their prey, that is:

(11) $$\Delta B_{i}\left(t\right)=B_{i}\left(t\right)\frac{tot\_biomass\_dead\_predators}{tot\_biomass\_prey}$$

This quantity can be translated into a consequent potential increase in the prey’s abundance, allowed by the decrease of its predators,

(12) $$\Delta X_{i}\left(t\right)=\frac{\Delta B_{i}\left(t\right)}{M_{i}}$$

and this increase can therefore be added to its carrying capacity, so that:

(13) $$K_{i}\left(t+1\right)=K_{i}\left(t\right)+10\times \Delta X_{i}\left(t\right)$$

for herbivores, fungivores, and bacterivores (first trophic level), and

(14) $$K_{i}\left(t+1\right)=K_{i}\left(t\right)+\Delta X_{i}\left(t\right)$$

for carnivores and omnivores (second trophic level). The multiplicative factor 10 in Equation (13) was inserted according to the Eltonian rule across adjacent trophic levels (Elton, 1927).

The new value for $K_{i}\left(t+1\right)$ will be inserted in the Equation (9), thus influencing the further dynamical evolution of the system. The same rule does not need to be applied if the decrease concerns the abundances of herbivores, fungivores, and bacterivores since these grazing species are according to the lemma only prey; therefore, they cannot induce variations in the carrying capacity of other species.

In order to quantify structural changes and to compare one single simulation to others, that is, the results of the simulations carried out by removing either guild, we introduce an *Alteration Index* (AI), defined for each ecosystem as follows:

(15) $$\text{AI}=\sum_{k}\frac{\left|X_{\mathrm{sk}}-X_{\mathrm{fk}}\right|}{X_{\mathrm{sk}}}=\sum_{k}\frac{\left|\Delta X_{k}\right|}{X_{\mathrm{sk}}}$$

where *X_sk_* and *X_fk_* are, respectively, the abundance of species *k*‐th calculated after 100 time steps, that is, in the steady state, and the abundance of the same species calculated at the end of the simulation. In other words, AI considers the sum of the absolute variations in abundance that the species undergo due to the forced removal of some other species, normalized with respect to their abundance in the steady state. Our AI is of particular interest as the stability of depaupered food webs remaining after deleting functional groups has not been examined systematically by Hunt and Wall (2002). These authors state that such a new food‐web stability may be critically important to ecosystem function, and therefore, we see AI as a measure of the alteration of the ecosystem due to the introduced perturbation. Note that here the summation includes only the guilds for which forced removal does not occur, since we are interested in quantifying only the direct effects of the perturbation (without including the perturbation itself).

## 3 COMPUTATIONAL RESULTS

Let us now discuss in detail the simulations performed in nine different scenarios: removing separately the five main guilds or removing four combinations of them (we are not aware of any study where a combination of functional guilds was removed, although independent cascading effects due to the loss of a single guild have been addressed by Hunt and Wall (2002)).

**FIGURE 1:**
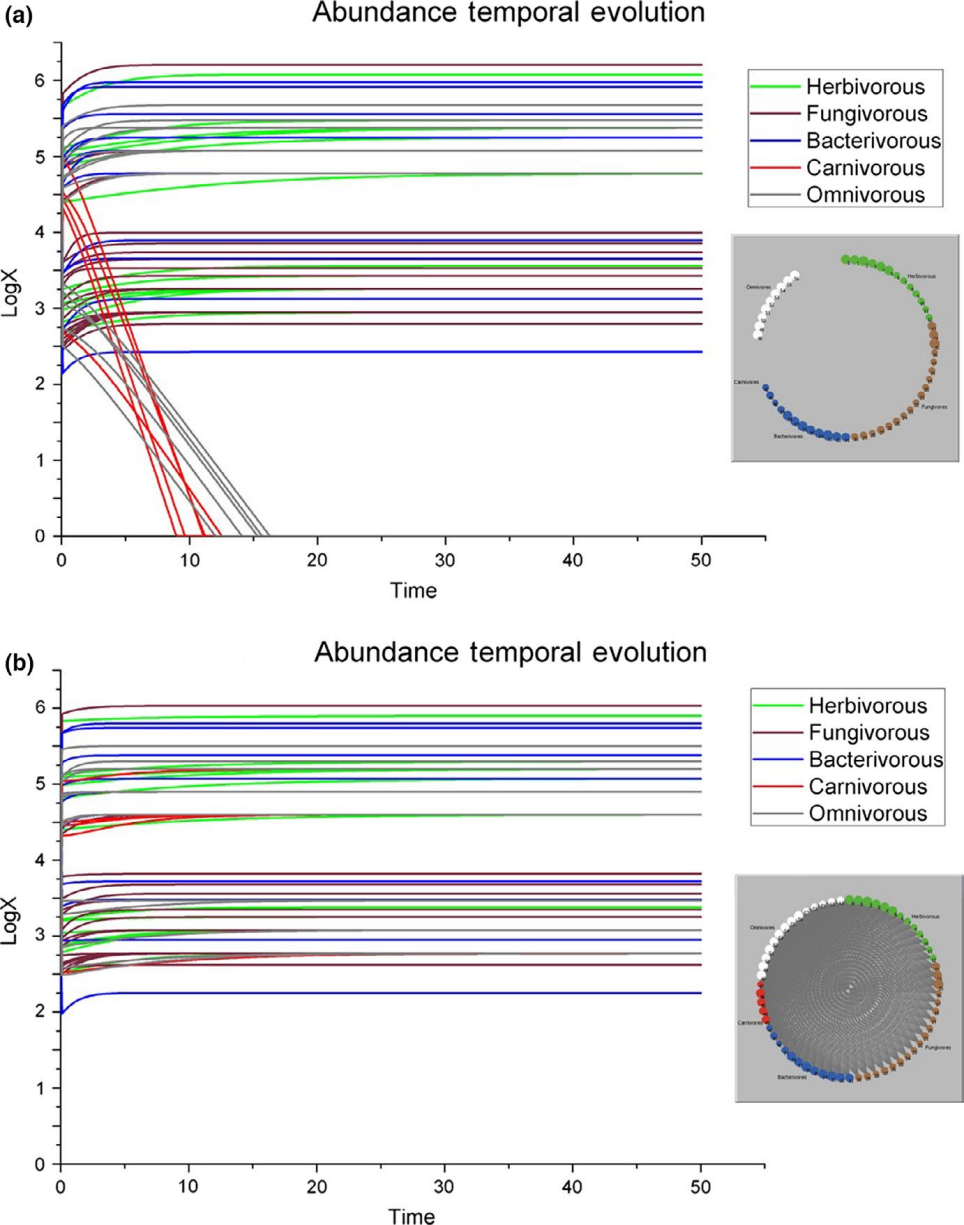
Graphic representation of the simulation results (a) without interaction (*β* = 0), upper panel, or (b) with interaction (*β* = 1), lower panel. Left, the temporal evolutions of species abundances are shown [50 time steps]. Right, the representation of the food web at the end of the simulation as interaction network of the soil invertebrates in the investigated area: Each line is an expected link between the organisms belonging to the first trophic level (primary consumers, being herbivorous, fungivorous, or bacterivorous invertebrates, or a combination of them, i.e., generalists) and the organisms belonging to the second trophic level (secondary consumers, either carnivorous or predatory omnivorous invertebrates)

In the simulation shown in Figure 1a, we set *β =* 0 in order to test the behavior of the system in the (unrealistic) scenario in which there were no interactions among species. As expected, species with *r_i0_ < 0* (i.e., the carnivorous and omnivorous species 57, 58, 59, 60, and 61) become extinct. The other species, instead, rapidly reach their own carrying capacity. On the other hand, in the simulation shown in Figure 1b, we set *β =* 1, that is, we consider the standard scenario in which there is interaction between species, but the system is not disturbed. In this case, all species reach their carrying capacity and the system goes rapidly in balance. This is the condition with which we have to compare all the scenarios, in which we always set *β =* 1, but the soil system is perturbed by the forced loss of entire guilds.

In summary, we will perform nine simulations differing one from each other concerning the functional guilds which were removed from the food web. We removed either one or more guilds (even an entire trophic level in the case of the removal of all omnivores and carnivores) to forecast an evolution of such an artificially depaupered food web.

**FIGURE 2:**
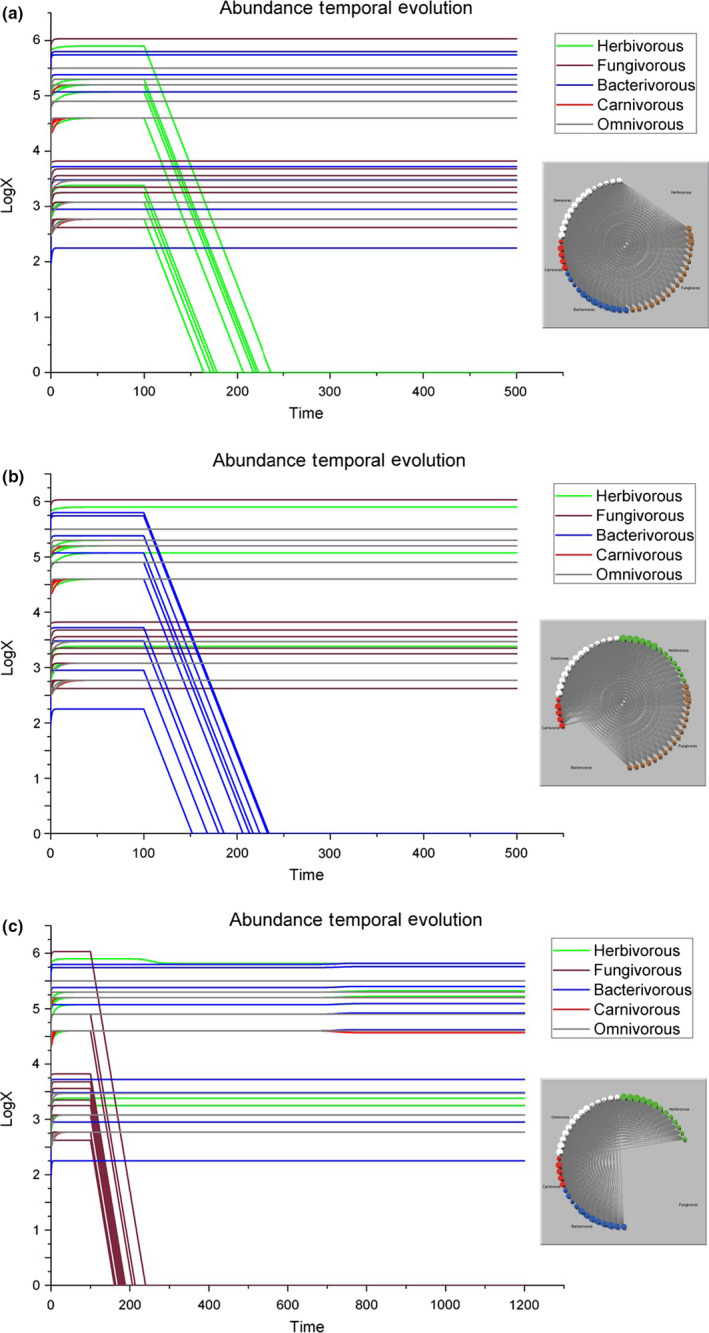
Graphic representation of the simulation results in the case all herbivores (a), bacterivores (b), or fungivores (c) are removed from the ecosystem. Left, the temporal evolutions of species abundances and right, the representations of the food web at the end of these three simulations [for the upper two panels, the first 500 time steps are shown; in the lower panel, fluctuations occur after 700 time steps]

**FIGURE 3:**
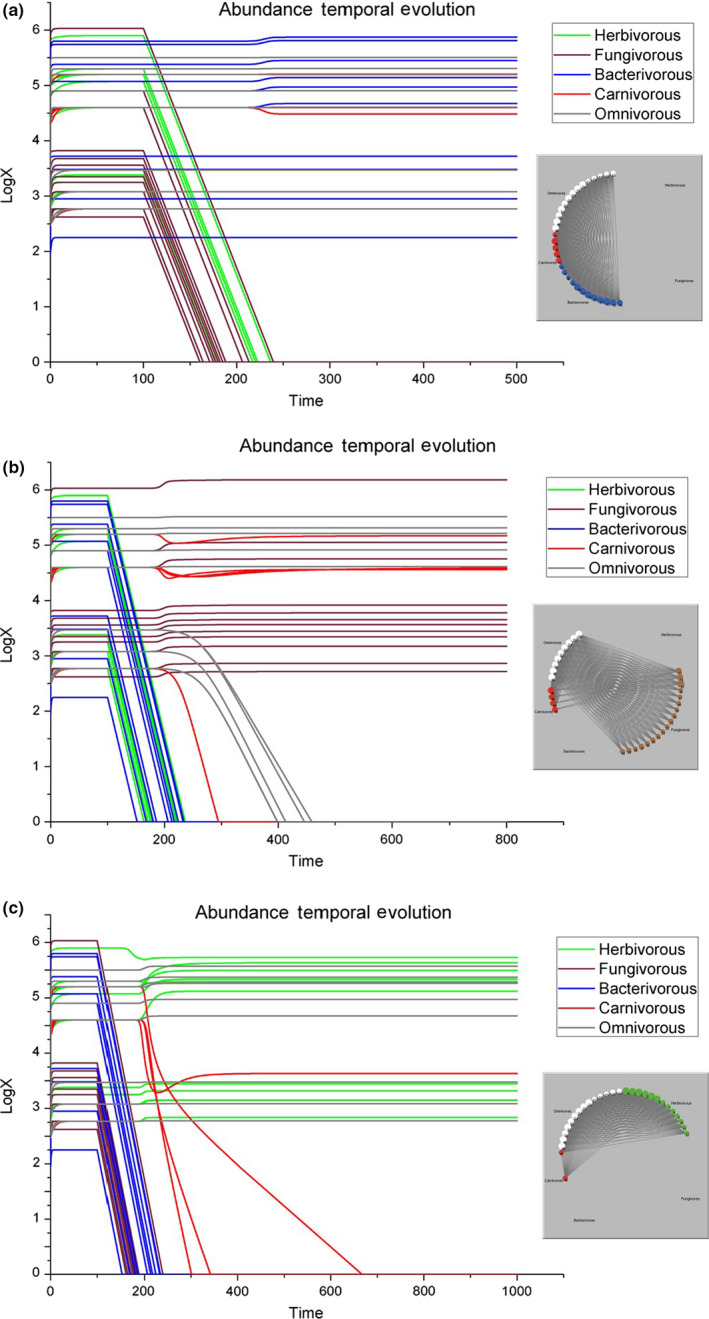
Graphic representations in the case all herbivores and fungivores (a), all herbivores and bacterivores (b), or all fungivores and bacterivores (c) are removed from the ecosystem. Left, the temporal evolution of species abundances and right, the representations of the food web at the end of the three simulations [note the different time steps on the horizontal axes]

**FIGURE 4:**
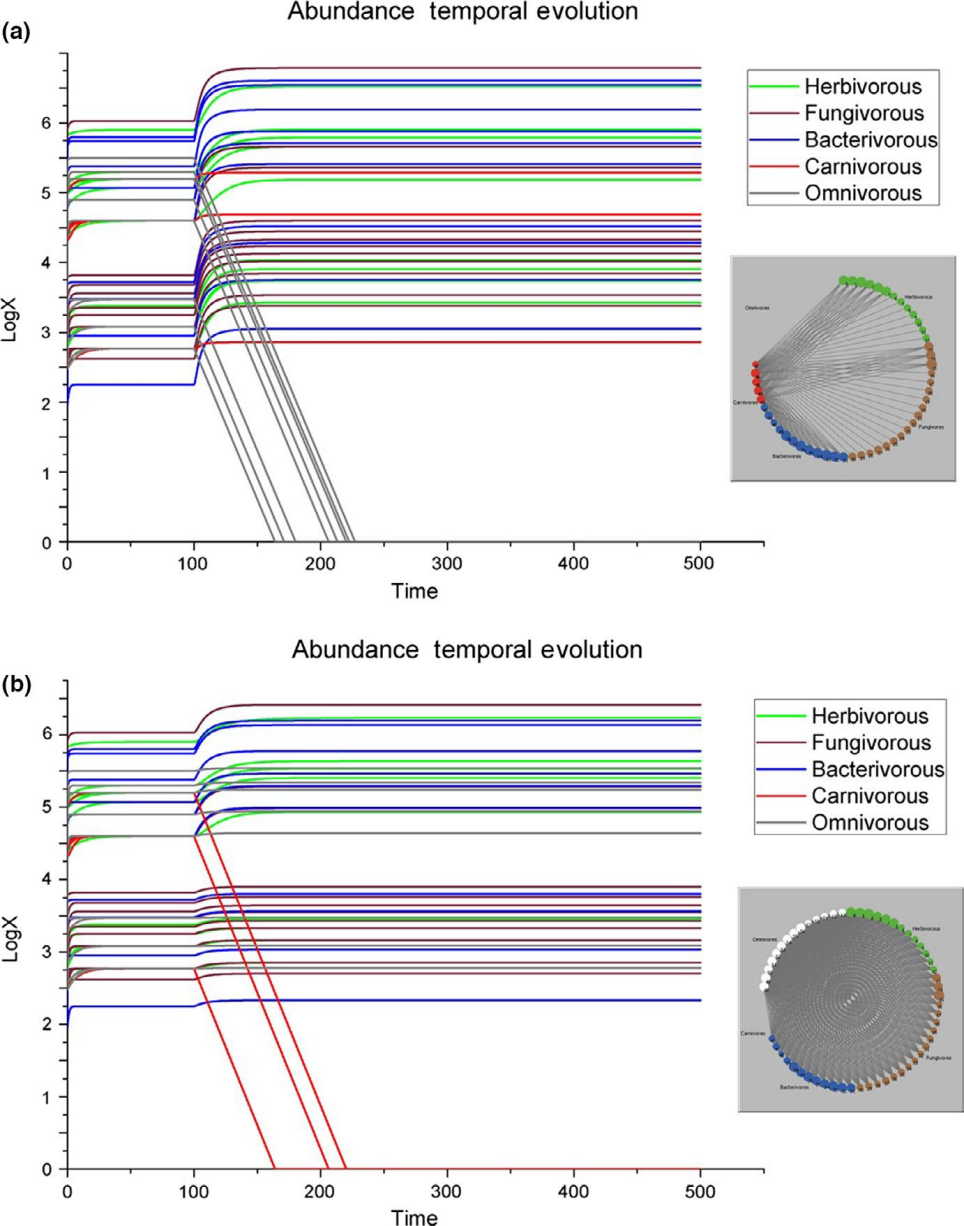
Graphic representation in the case all omnivores (a) or carnivores (b) are removed from the ecosystem. Left, the temporal evolutions of species abundances [500 time steps] and right, the representations of the food web at the end of these two simulations

**FIGURE 5:**
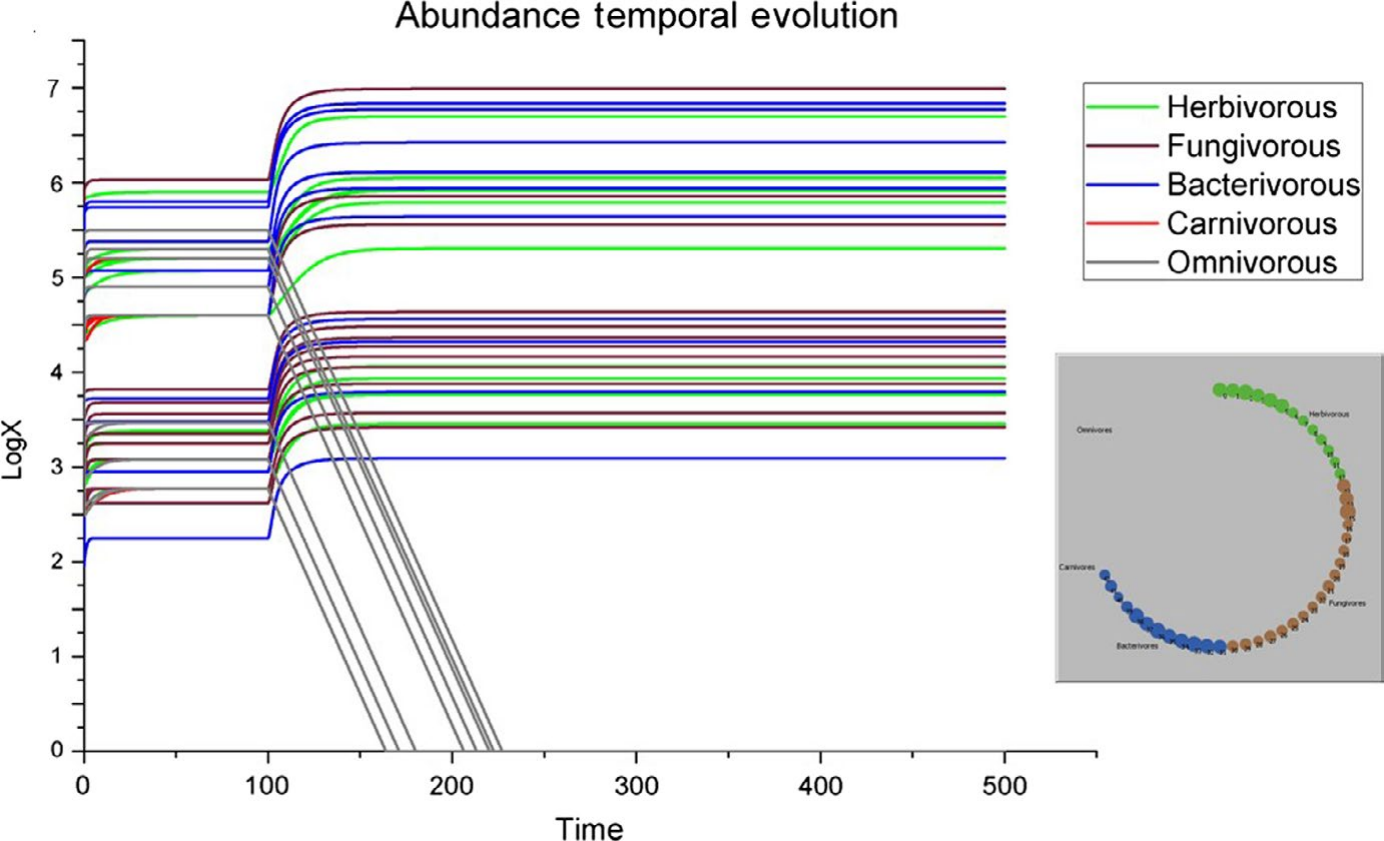
Graphic representation in the case all omnivores and carnivores are removed together from the ecosystem. Left is shown the temporal evolution of species abundances [500 time steps] and right the representation of the food web at the end

- *Removal of either all the herbivorous species together or all the bacterivorous species together*. The soil system does not seem to be affected by any of these disturbances, and all other species settle at their carrying capacity (Figure 2a and b), and the alterations of the food‐web architectures, as computed by Equation (15), are in both scenarios statistically undistinguishable from 0. These comparable results for such different scenarios are rather surprising, as the lumped biomass of the bacterivores is 1.58 times larger than the lumped biomass of the herbivores (10^5.62^ versus 10^5.42^ log[μg/m^2^ dry weight], respectively), in contrast to the removal of all the fungivorous species (sharing a much higher lumped biomass of 10^6.19^ log[μg/m^2^ dry weight]), a deletion that really influences the numerical abundances of other species (next paragraph). A possible biological explanation is, on the one hand, the specialization of grazing invertebrates, as herbivores and bacterivores have evident morphological adaptations to attack plant roots and ingest bacterial cells (Yeates, Bongers, De Goede, Freckman, & Georgieva, 1993), in contrast to larger consumers like fungivore oribatids (Figure 2c) which can handle many more different resources than smaller consumers like bacterivore nematodes (Figure 2b). On the other hand, the relative energetic contribution in terms of flux is on average the highest for herbivorous microarthropods and bacterivorous enchytraeids (Mulder et al., 2008), and therefore, we would have expected stronger cascading effects.
- *Removal of all the fungivorous species*. In this case, being the fungivorous Tylenchidae (node 15) the most abundant species of the entire system, its removal causes a slight decline of the second most abundant species of the system, the herbivorous *Helicotylenchus* (node 2), which decreases from 10^5.9^ to 10^5.82^. A possible explanation of this unexpected correlation between a fungivorous nematode species and a herbivorous nematode species could be due to an indirect cascading effect where the removal of the most abundant species forces a shift in the prey–predator relationships (AI = 0.94). Consequently, after about 400 time steps with respect to the diminishing herbivorous species at node 2, a minimal decrease in the abundance of carnivorous *Anatonchus* (node 43 of Table S1) from 10^4.6^ to 10^4.56^ is observed (Figure 2c), as resources do not seem to be sufficient. *Anatonchus* is well known to be a stress‐resistant nematode, capable to survive and dominate in hostile environments (Fiscus & Neher, 2002; Neher, Wu, Barbercheck, & Anas, 2005).
- *Removal of all the herbivorous and fungivorous species together*. The abundance of the carnivorous nematode *Anatonchus* (node 43 of Table S1) decreases from 10^4.6^ to 10^4.48^ and, as a consequence, a slight increase is also observed in some species of bacterivorous nematodes and enchytraeid worms (Figure 3a), and the alteration is correspondingly low (AI = 1.82). Being *Anatonchus* the most abundant predatory nematode, the lack of herbivorous and fungivorous prey not only affects this predatory species but also enhances the occurrence of bacterivorous prey at a lower trophic level.
- *Removal of all the herbivorous and bacterivorous species together*. The carnivorous mite *Dendrolaelaps* (node 47 of Table S1) and the truly omnivorous mites (nodes 57–61) become extinct. Other carnivores (43–46) succeed in adapting their population size to the much fewer resources available. There is also a slight increase in fungivores and in the remaining omnivores (Figure 3b). At the end, this simulation is resulting in a strong re‐assemblage of the food web with an *AI* equal to 11.53 and the fluctuations in the numerical abundances of so many species were rather unexpected given the lack of response of the food web to the removal of herbivores alone (Figure 2a) or bacterivores alone (Figure 2b). In short, the sum of the effects due to the removal of both guilds was highly relevant even if the separate removal of these two guilds did not influence our web.
- *Removal of all the fungivorous and bacterivorous species together*. Carnivores 44, 45, and 46 become extinct, but even the abundance of second most abundant species, the herbivorous *Helicotylenchus* (node 2), decreases. There is still a slight increase in herbivorous and omnivorous species (Figure 3c); in fact, AI reaches 12.97. Only the carnivores 43 and 47 seem to succeed in adapting their number to the fewer resources available. As aforementioned, *Anatonchus* is well known as a stress‐resistant nematode, and therefore, its successful performance is expected. The same holds for the mite *Dendrolaelaps*, because as generalist its trophic niche will be much larger than that of other more specialized carnivores. As in the previous figure, the effect due to the removal of two guilds at the same time was really relevant, although it should be mentioned that these effects are likely due to the removal of fungivores (Figure 2c) more than the removal of bacterivores (Figure 2b). In any case, the trophic effects due to the combined removal of microbivores in Figure 3c were opposite for the first trophic level (mostly increase in herbivorous species) and the second trophic level (mostly decrease in carnivorous species).
- *Removal of either all the omnivorous species or all the carnivorous species*. Omnivores are known to have the capability to modify their feeding behavior based on the resource profitability, either by switching prey species (Holt & Polis, 1997) or by adjusting the relative proportion of each prey across different guilds (for instance, preying on the most abundant basal species, like in our case fungivorous 15, herbivorous 2, and bacterivorous 33). Therefore, omnivores and food‐web architecture are closely linked to each other and a high degree of omnivory mostly stabilizes the web structure (Holt & Polis, 1997, and Kratina, LeCraw, Ingram, & Anholt, 2012, respectively). However, our Figure 4a clearly shows the reduced exploitation of the soil system by the omnivores once the entire functional guild has been removed. This destabilizing system is assessed by a remarkably high food‐web architecture alteration (AI = 195.75), the highest alteration due to the deletion of a single guild. In contrast, Figure 4b shows that the removal of only the carnivorous species causes only a disproportionally small impact on the food‐web architecture (AI = 29.41).
- *Removal of all the invertebrates at the second trophic level (omnivorous and carnivorous species)*. Omnivores and carnivores ate the producers in proportion to their biomass, with omnivores sharing no evident random preference. As expected, an increase in the abundance of all other species at the first trophic level is observed, showing the response of the system to the lack of top‐down control (Figure 5). This scenario is the one with the highest alteration value despite it does not lead to the secondary extinction of any species because the entire upper trophic level was removed. In fact, the removal of omnivores and carnivores causes a significant increase in their prey according to the Equation (13) and the total alteration due to the removal of omnivores and carnivores together is much more than the sum of the single alterations due to the removal of either the omnivores or the carnivores (261.41 > 195.75 + 29.41 = 225.16). With an additional alteration of 16% of the food‐web architecture, we can only conclude that omnivory strongly enhances the flux of nutrients between the soil invertebrates belonging to the first trophic level and those belonging to the second trophic level.

## 4 CONCLUSIONS

Natural ecosystems are increasingly subjected to severe stress events due to global warming, deforestation, and resource depletion, as evidenced by numerous studies, for example, Hunt and Wall (2002), Malhi et al. (2008), Barnosky et al. (2011); Barnosky et al. (2012), Ceballos et al. (2015), but the contribution of particular species to the compensation and the community resistance after the extinction of other co‐occurring species is uncertain (Ives & Cardinale, 2004; Strona & Bradshaw, 2018). Therefore, the study of food webs is of fundamental ecological importance, as webs define the structure of the ecosystem and determine its properties, including its stability to stress. Any ecosystem is stable if the system opposes to its disintegration when subject to various types of disturbances that can cause the extinction of species. Following a numerical approach able to combine both dynamical and topological aspects of the problem, in this paper we quantified the reaction of a real density‐driven ecological network of soil invertebrates to different perturbative scenarios by means of extended simulations realized within a fully programmable agent‐based environment. If we consider the average number of species per guild as functional redundancy, the removal of all fungivorous and bacterivorous species lessens 60% of redundancy of our food web in time. Assuming like Borrvall, Ebenman, and Jonsson (2000) and Kratina et al. (2012) that a major effect of omnivory is to lessen the risk of species extinctions following the loss of a herbivore, it is surprising that so many omnivores got extinct. However, missing top‐down control by secondary consumers in general, and by omnivores in particular, independently increased the primary consumers and confirmed a recent meta‐analysis (Mancinelli & Mulder, 2015). These authors concluded in fact that omnivores, either alone or with predators, exerted a much stronger negative effect than solely predators in terrestrial systems. Seen that the species loss can be assessed when guilds disappear, this method visualizes alterations in the food‐web architecture.

## CONFLICT OF INTEREST

The authors declare no competing interest.

## AUTHOR CONTRIBUTION

**Erminia Conti:** Conceptualization (lead); Formal analysis (supporting); Methodology (equal); Resources (equal); Supervision (equal); Validation (equal); Writing‐original draft (equal); Writing‐review & editing (supporting). **Letizia Stella Di Mauro:** Formal analysis (lead); Investigation (supporting); Software (supporting); Visualization (equal); Writing‐original draft (equal). **Alessandro Pluchino:** Conceptualization (equal); Formal analysis (equal); Investigation (equal); Project administration (equal); Software (equal); Supervision (equal); Writing‐original draft (lead). **Christian Mulder:** Conceptualization (equal); Data curation (lead); Investigation (equal); Methodology (equal); Resources (equal); Supervision (equal); Writing‐original draft (equal); Writing‐review & editing (lead).

## Supporting information

Supplementary Material

Fig S1

## Data Availability

All trophic links are archived in the Wiley Repository (Mulder and Elser (2009): their table S2) at https://onlinelibrary.wiley.com/doi/full/10.1111/j.1365‐2486.2009.01899.x
