# Supplementary material for: Testing for top‐down cascading effects in a biomass‐driven ecological network of soil invertebrates
Source: Ecol Evol. 2020 Jun 18;10(14):7062–72. doi: 10.1002/ece3.6408 (PMC7391537; doi:10.1002/ece3.6408)
Supplement: Supplementary file 1 — Supplementary Material [file ECE3-10-7062-s001.docx]

**SUPPLEMENTARY MATERIAL**

**APPENDIX**

In the simplest population growth model (Neal, 2019), the rate of population increase is proportional to the population size at any time, assuming unlimited resources. Let us denote by *N(t)* the population at time *t* and by *r* a positive constant.

Then $\frac{dN}{dt}=rN$ and by integration $N\left( t \right)=N_{0}e^{rt}$ (S1)

where *N*_0_ denotes the population at the time *t = 0* and *r* is the intrinsecal rate of natural increase between rate of birth *b* and death rate *d*. But in the real world, resources are not unlimited. The consequent reduction of the resources affects *r* and produces, obviously, an increase of the mortality rate and a decrease of the natality rate. The variation of *b* and *d* as function of *N* can be assumed as linear: $b=b_{0}-aN$ and $d=d_{0}+ cN$, where *b*_0_ and *d*_0_ are the values respectively of the birth rate and of the death rate when *N* is close to 0. This model can be written as:

$\frac{dN}{dt}=\left[ \left( b_{0}-aN \right)-\left( d_{0}+cN \right) \right]N$ (S2)

that describes intra-specific regulation of the population. When *b > d* population increases with a slower growth rate for increasing value of *N*. When *b < d* the growth becomes negative and population decreases. When *b = d* the abundance of the population reaches a stationary state corresponding to its maximum sustainable value, also called carrying capacity *K* of the system. This value can be obtained by putting $\frac{dN}{dt}=0$ in equation (S2), which gives the two fixed points of the dynamics, *N = 0* (repulsive) and *N = K* (attractive), as: $K=\frac{b_{0}-d_{0}}{a+c}$ (S3)

It is likely important to state that we did not assume that all species have the same carrying capacity as in other simulations (Gross and Cardinale, 2005), but species-specific *K* values. Using their carrying capacity, equation (S2) takes the well-known form of the logistic equation (Verhulst, 1838) that describes the sigmoidal growth of a population when prey/resources are limited:

$\frac{dN}{dt}=rN\left( 1-\frac{N}{K} \right)$ (S4)

with *r = b_0_ − d_0_*. Within each guild, we derived the *r* values for the functional groups shown in Table S1 from Moore et al. (1993) and De Ruiter et al. (1995).

Equation (S4) has two components: an exponential term (*rN*), which is important for small *N* values, and a second one $\left( 1-\frac{N}{K} \right)$ that reduces the population growth when approaching the environmental-driven carrying capacity. Given our lemma of unlimited basal resource supply of bacteria, fungi and roots (in contrast to limited non-basal resources, see Kondoh, 2005), the main assumptions are:

- In the absence of predators, the population *X*_1_ of the prey would grow proportionally to its size,

$\frac{{dX}_{1}}{dt}=A_{1}X_{1}$ (S5)

being *A*_1_ > 0 the growth rate of the prey. So in this case a Malthusian growth of the prey occurs.

- In the absence of prey, the population *X*_2_ of the predator would decline proportionally to its size,

$\frac{{dX}_{2}}{dt}=A_{2}X_{2}$ (S6)

being *A*_2_ < 0 the predators’ death rate, meaning extinction of that population.

- When both predators and prey are present, to the previous terms is added a quantity representing the effect of the predation, that is a decrease in the prey population and a growth in the predator population:

$\frac{{dX}_{1}}{dt}=A_{1}X_{1}+A_{12}X_{2}X_{1} \frac{{dX}_{2}}{dt}=A_{2}X_{2}+A_{21}X_{1}X_{2}$ (S7)

being *A*_12_ < 0 and *A*_21_ > 0, respectively, the negative and the positive growth rates for prey and predators.

REFERENCES

De Ruiter, P.C., Neutel, A.-M. & Moore, J.C. (1995). Energetics, patterns of interaction strengths, and stability in real ecosystems. *Science*, 269, 1257-1260.

Gross, B., Cardinale, B.J. (2005). The functional consequences of random vs. ordered species extinctions. *Ecol. Lett.*, 8, 409-418.

Kondoh, M. (2005). Linking flexible food web structure to population stability: A theoretical consideration on adaptive food webs. In: De Ruiter, P.C., Wolters, V. & Moore, J.C. (eds.). Dynamic Food Webs – Multispecies assemblages, ecosystem development and environmental change. Theoretical Ecology Series, Academic Press, Burlington, MA, pp. 101-113.

Moore, J.C., De Ruiter, P.C. & Hunt, H.W. (1993). Influence of productivity on the stability of real and model ecosystems. *Science,* 261, 906-908.

Neal, D. (2019). *Introduction to Population Ecology*. Cambridge University Press, Cambridge, UK.

Verhulst, P.-F. (1838). Notice sur la loi que la population poursuit dans son accroissement. *Correspondance Mathématique et Physique*, 10, 113-121.


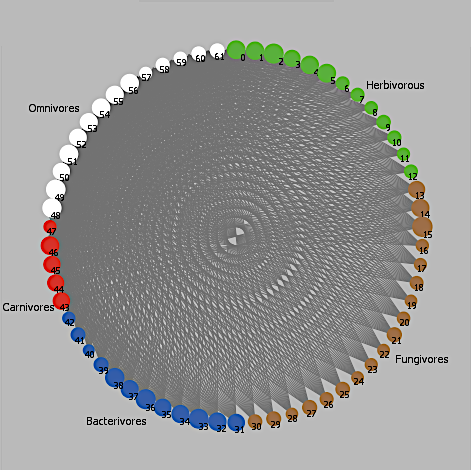


Online Figure S1. A sketch of the food web network with 62 nodes/species organized in groups of different colours (see Table S1) and placed in a circular layout. Directed links represent the prey/predator connections, going from the prey node to the predator node.


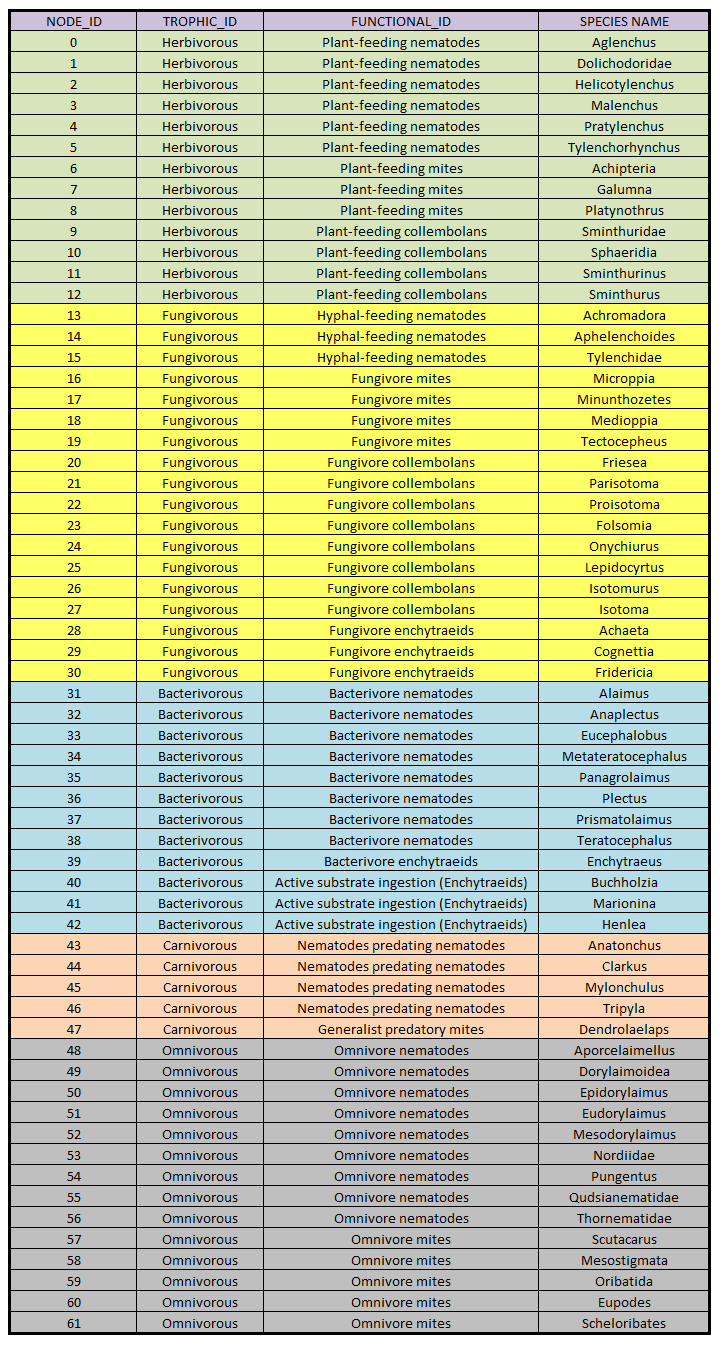


Online Table S1. Trophic ID, functional ID and name of the taxa (mostly families or genera, hereafter called “species”) corresponding to the nodes of the network shown in Figure S1.
