# Supplementary figures and images for: Testing for top‐down cascading effects in a biomass‐driven ecological network of soil invertebrates
Source: Ecol Evol. 2020 Jun 18;10(14):7062–72. doi: 10.1002/ece3.6408 (PMC7391537; doi:10.1002/ece3.6408)

## Slide 1
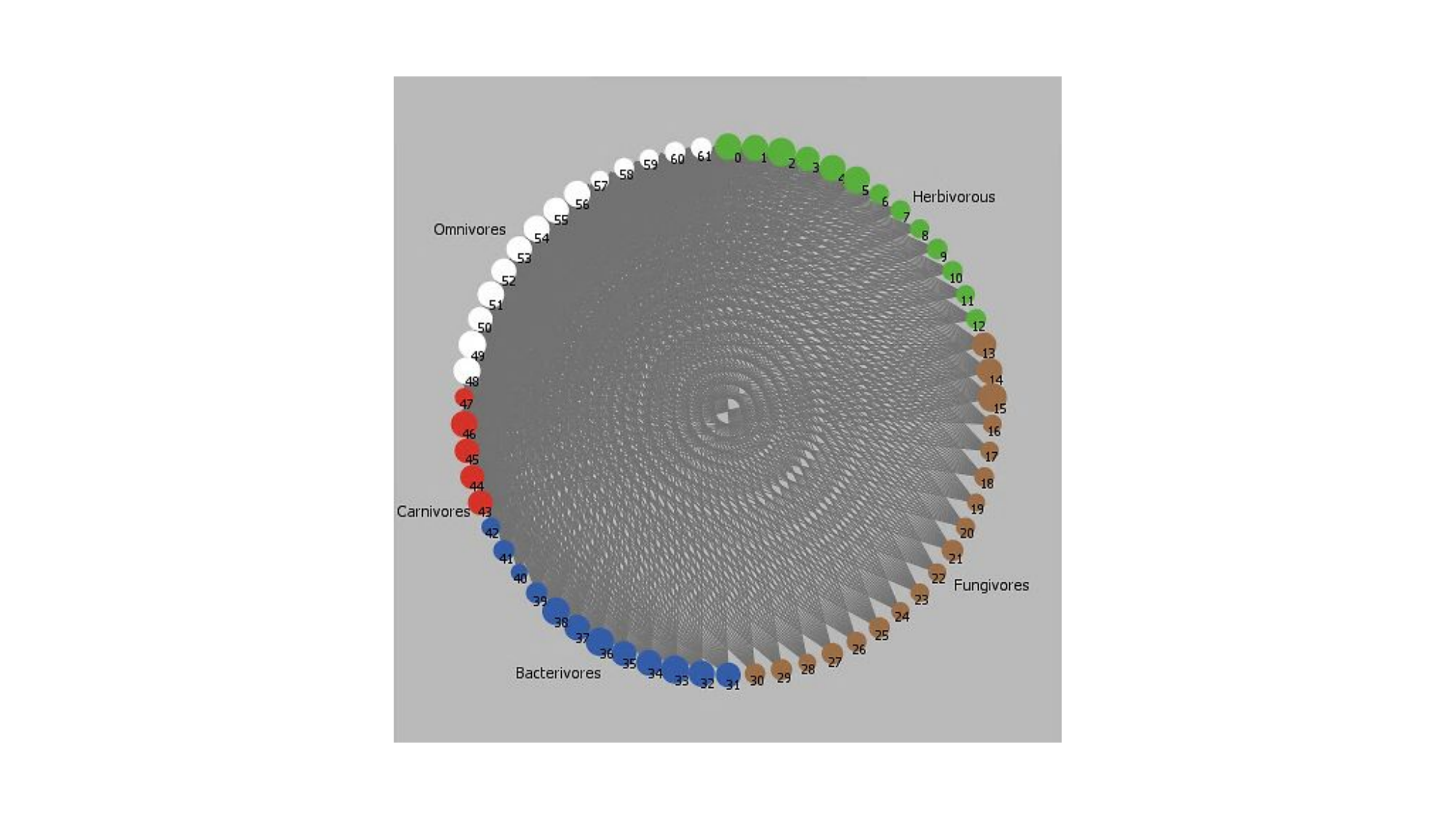

Supplement: Supplementary file 2 — Fig S1 [file ECE3-10-7062-s002.pptx]
